# Targeting glioma-associated microglia and macrophages: a new frontier in glioblastoma immunotherapy

**DOI:** 10.3389/fimmu.2025.1726440

**Published:** 2025-12-17

**Authors:** Bingyang Wang, Cong Li, Jiatong Gu, Xiaojie Wang, Mingjuan Xun, Bin Jiang, Jun Yan

**Affiliations:** 1 Department of Neurology, Shenzhen Qianhai Shekou Free Trade Zone Hospital, Shenzhen, China; 2 Beijing Institute of Brain Disorders, Laboratory of Brain Disorders, Ministry of Science and Technology, Collaborative Innovation Center for Brain Disorders, Capital Medical University, Beijing, China

**Keywords:** glioblastoma, glioma-associated microglia/macrophages, immune evasion, immunotherapeutic strategies, polarization, tumor microenvironment

## Abstract

Glioblastoma (GBM), the most aggressive and lethal subtype of glioma, remains therapeutically intractable despite advances in surgical and chemo-radiotherapy interventions. The highly immunosuppressive tumor microenvironment (TME) contributes significantly to treatment resistance and tumor recurrence. Among the predominant immune constituents, glioma-associated microglia and macrophages (GAMs) constitute a major cellular compartment, exerting profound influence on tumor progression, immune evasion, angiogenesis, and therapeutic response. These myeloid populations, derived from both yolk sac–origin microglia and bone marrow–derived macrophages, exhibit remarkable functional plasticity and are actively recruited, polarized, and reprogrammed by tumor-intrinsic and environmental cues. Recent studies have elucidated a range of molecular pathways, including chemokine signaling, metabolic reprogramming, and epigenetic modulation, that govern GAM behavior and sustain their tumor-supportive phenotype. Therapeutic strategies targeting GAM recruitment, depletion, or functional re-education toward an anti-tumor state are emerging as promising adjuncts to conventional and immune-based therapies. This review comprehensively explores the ontogeny, regulatory networks, and pathological roles of GAMs in GBM, with particular emphasis on novel immunotherapeutic approaches, including CSF-1R blockade, nanoparticle-mediated reprogramming, and oncolytic virotherapy. A deeper understanding of GAM–TME interactions will be critical to overcoming immunotherapy resistance and advancing precision immunomodulation in GBM.

## 1 Introduction

Gliomas represent the most prevalent form of malignancy within the central nervous system (CNS), contributing to 80% of all malignant brain cancers (1). Among the histological subtypes, glioblastoma (GBM) stands out as the most aggressive variant, accounting for approximately 70–75% of glioma cases (2). Despite aggressive multimodal therapy—comprising extensive surgical resection followed by radiochemotherapy—the median survival for individuals diagnosed with GBM remains under 20 months (3). Furthermore, disease recurrence occurs in nearly 80% of patients, predominantly within or proximal to the original surgical margin (4). These bleak clinical outcomes highlight an urgent demand for more effective treatment modalities, with immunotherapeutic approaches emerging as a particularly promising direction in GBM care.

The tumor microenvironment (TME) has been increasingly recognized not only as a central orchestrator of tumor development and progression but also as a critical contributor to the phenotypic and molecular heterogeneity observed within GBM (5, 6). A key component of this microenvironment is the GAMs, which constitute the dominant immune cell population in gliomas. Their infiltration correlates positively with tumor grade, often making up 30–50% of the total tumor cellular content (6). GAMs exert multifaceted effects on the TME, significantly influencing tumor growth, immune suppression, and therapeutic resistance (7, 8). Accumulation of GAMs is strongly linked with glioma advancement and is indicative of unfavorable prognosis in GBM patients, underscoring their potential as crucial targets for immunomodulatory interventions (9). This review delineates the developmental origins of GAMs, outlines the signaling axes governing their recruitment and polarization, and explores their functional contributions to glioma biology. Particular focus is placed on recent progress in therapeutic strategies that aim to manipulate GAMs activity to enhance the efficacy of GBM treatments.

## 2 Microglia and macrophages in glioblastoma

In glioblastoma, the tumor microenvironment is marked by minimal T cell presence but a pronounced enrichment of GAMs, which collectively constitute over 30% of the infiltrating immune population within the neoplastic niche (10). This population includes both infiltrating macrophages, derived from circulating monocytes, and resident microglial cells. Monocyte-derived macrophages originate in the bone marrow, where they differentiate in response to cytokine cues before migrating into peripheral tissues (11). Conversely, microglia stem from yolk sac–derived erythromyeloid precursors and undergo lineage specification regulated by defined transcriptional programs, ultimately settling in specific compartments of the central nervous system during development (12). Importantly, GAMs exhibit the ability to self-renew and engage in competitive interactions for spatial occupancy within the TME (13). In both primary and relapsed GBM lesions, particularly under hypoxic conditions, the majority of GAMs are derived from microglia rather than from monocytes (14).

### 2.1 Recruitment of microglia and macrophages

The glioblastoma tumor microenvironment harbors a dense network of chemokines and inflammatory mediators that orchestrate the recruitment of GAMs (15, 16). Substantial progress has been made in delineating the molecular underpinnings of this process. Aberrant metabolic activity within tumor cells not only alters intrinsic signaling but also remodels the surrounding stroma (17, 18). For instance, the metabolic byproduct kynurenine (Kyn), produced during GBM-associated metabolic rewiring, activates aryl hydrocarbon receptor (AhR) signaling in GAMs (19, 20). This activation triggers upregulation of chemokine (C-C motif) ligand 2 (CCL2), which facilitates the directed migration of GAMs toward the tumor site (19). In addition to this ligand–receptor cascade, several signaling axes have been implicated. The guidance molecule SLIT2 engages roundabout receptors ROBO1 and ROBO2, whose expression on target cells mediates their chemoattraction. In GBM, SLIT2–ROBO interactions promote GAMs infiltration via activation of the PI3K pathway (21). Importantly, downstream of PI3K, activation of small Rho GTPases such as Rac1 and Cdc42 orchestrates actin cytoskeletal remodeling, lamellipodia and filopodia formation, and directional migration of GAMs (22, 23). These cytoskeletal changes are critical for enabling GAMs to traverse the dense extracellular matrix and reach tumor foci. Therefore, the PI3K–Rac1/Cdc42 axis represents a key mechanistic bridge linking chemotactic signaling to the physical motility of glioma-infiltrating myeloid cells (24). Moreover, the receptor tyrosine kinase mesenchymal–epithelial transition factor (MET) is notably upregulated within the TME of secondary GBM, and has been shown to initiate the STAT4–PD-L1 signaling cascade in primary GBM, thereby enhancing GAMs infiltration and contributing to immune escape mechanisms (25, 26). Epigenetic alterations in glioma cells profoundly influence the immunological landscape. Among these, N6-methyladenosine (m6A), a prevalent RNA epigenetic modification in eukaryotic cells, is subject to dynamic regulation in response to hypoxic stress (27). GBM cells elevate expression of the demethylase ALKBH5, significantly increasing GAMs accumulation in xenograft models (28, 29). Likewise, glioma stem cells (GSCs), through persistent transcriptional and epigenetic remodeling, activate gene expression programs characteristic of bone marrow-derived lineages, which in turn amplify GAMs recruitment (30).

### 2.2 Polarization of GAMs

GAMs exhibit functional plasticity, shifting between pro-inflammatory, tumor-suppressive M1-like states and anti-inflammatory, tumor-supportive M2-like phenotypes (31). These subsets are not rigidly fixed and can transition bidirectionally depending on local cues. The M2-like subset comprises several variants—namely M2a, M2b, M2c, and M2d—that span a spectrum of functional states (32, 33). In the process of acquiring the M2 phenotype, GAMs secrete immunomodulatory and tumor-promoting factors such as TGF-β, epidermal growth factor (EGF), IL-10, and the proteolytic enzymes MMP-2 and MMP-9, thereby reinforcing an immunosuppressive tumor milieu within the glioblastoma microenvironment (31, 34). Among these M2 subtypes, M2a macrophages—typically induced by IL-4/IL-13—are associated with tissue repair and wound healing, whereas M2d macrophages, often driven by IL-6 and adenosine signaling, promote tumor angiogenesis and immunosuppression (35). Recent single-cell transcriptomic and spatial profiling studies of human GBM specimens have indicated a predominance of M2d-like polarization signatures within the GAM compartment, particularly localized around perivascular niches, suggesting their functional relevance in supporting neovascularization and immune evasion in GBM (36–38). In contrast, M2a-like signatures appear more spatially restricted and are enriched in regions undergoing tissue remodeling or repair post-therapy, indicating a context-dependent distribution of M2 subtypes (38).

Cytokines are pivotal regulators orchestrating GAMs polarization in the tumor microenvironment (39). Evidence from both murine glioma models and human GBM specimens reveals that IL-33 enhances the expression of M2-associated markers, thereby skewing GAMs toward an M2-biased profile. Conversely, genetic ablation of IL-33 compromises this polarization trajectory (40). The IL-6 and IL-6R axis also exerts a significant influence. GBM-infiltrating GAMs expressing β-site amyloid precursor protein cleaving enzyme 1 (BACE1) or T cell immunoglobulin and mucin-domain containing-3 (TIM-3) engage IL-6R, triggering downstream signaling pathways that perpetuate their pro-tumorigenic and anti-inflammatory state. Notably, inhibition of BACE1 pharmacologically has been shown to redirect GAMs toward a tumor-restraining phenotype. Additionally, IL-6R blockade has been demonstrated to impede tumor progression *in vivo* (41, 42). BACE1 also modulates the polarization of GAMs through activation of the JAK/STAT3 signaling pathway, a critical driver of immunosuppressive mechanisms in glioma. BACE1 activation has been linked to JAK/STAT3 pathway activation, which enhances the M2-like polarization of GAMs (43). This pathway promotes immune suppression by upregulating the secretion of cytokines such as IL-10 and TGF-β, and by inhibiting the cytotoxic functions of tumor-infiltrating immune cells (44, 45). In particular, the activation of JAK/STAT3 by BACE1 contributes to a feedback loop that perpetuates a pro-tumorigenic and immune-evasive environment within the glioma TME (41, 46). Furthermore, chemotherapeutic agents such as temozolomide (TMZ), the standard treatment for GBM, may also modulate GAMs phenotypic plasticity. Certain TMZ-responsive long noncoding RNAs have been implicated in driving microglial polarization toward the M2 state, thereby fostering drug resistance (46). In contrast, GBM cells treated with TMZ can release high-mobility group box 1 (HMGB1), which activates the NF-κB–NLRP3 inflammasome signaling axis in GAMs, thus promoting their differentiation into the M1 subtype (47, 48).

## 3 Functions of GAMs

### 3.1 Enhancement of glioma cell growth and infiltrative potential

Within the tumor microenvironment, metabolic rewiring serves as a critical determinant of glioma cell proliferation and invasiveness (49, 50). Among glioma-associated GAMs, those exhibiting an M2 phenotype secrete IL-1β, which activates protein kinase δ through the PI3K cascade (51, 52). This signaling leads to phosphorylation of glycerol-3-phosphate dehydrogenase at threonine 10, subsequently boosting glycolytic flux and promoting tumor cell proliferation (51). Moreover, exosomes released by glioblastoma cells (GBex) have been shown to convert M1 macrophages toward a tumor-supportive phenotype and reinforce M2-like characteristics (53, 54). These GBex-educated GAMs secrete vesicles enriched with arginase-1 (ARG-1), a metabolic enzyme that further drives glioma cell propagation (55, 56). Chemokine-mediated signaling within the TME also governs tumor invasion by facilitating immune cell trafficking and directly influencing cancer cell behavior. For instance, CCL8, abundantly produced by GAMs, enhances glioma cell pseudopod extension and interacts with CCR1 and CCR5 receptors on tumor surfaces (57, 58). This ligand-receptor engagement triggers ERK1/2 phosphorylation, potentiating cellular invasiveness. In parallel, GAMs-derived CCL5 stimulates glioma motility and matrix degradation by activating MMP-2 through a calcium-dependent pathway (57, 59). These findings delineate a multifaceted regulatory axis wherein GAMs, via metabolic and chemokine-driven mechanisms, accelerate glioma progression and invasion.

### 3.2 Tumor angiogenesis facilitation

Neovascularization plays a fundamental role in sustaining tumor expansion, with endothelial cell activation acting as a key initiating event in this process (60, 61). Glioblastoma-derived IL-8 and CCL2 chemokines can activate glioma-associated GAMs, which, in turn, secrete TNF-α, thereby triggering gene expression programs in ECs characteristic of an activated state (62, 63). In a murine glioblastoma setting, administration of the bevacizumab analog B20.4.1.1 was associated with heightened TNF-α release by GAMs and increased endothelial activation—findings that may underlie the observed inefficacy of anti-angiogenic strategies in glioblastoma treatment (63, 64). Vascular endothelial growth factor (VEGF) is widely recognized as a core component of the pro-angiogenic signaling milieu. In glioblastoma cells deficient in the tumor suppressor PTEN, aberrant activation of the AKT–CSK9β–IRF9 axis promotes the overproduction of galectin-9 (65, 66). This lectin interacts with the Tim-2 receptor on GAMs, enhancing their M2-like polarization, which subsequently leads to VEGF-A secretion and stimulation of neovascular formation to support glioma progression (67). Furthermore, recent evidence has revealed that GAMs play an essential role in the modulation of angiogenesis via the activation of intracellular calcium flux (9, 68, 69). GAM-derived signals, particularly TNF-α, activate the phospholipase C (PLC) pathway, which triggers calcium influx and activates protein kinase C (PKC) and calcium/calmodulin-dependent protein kinase II (CaMKII) (59, 63). This signaling cascade reinforces the notion that GAMs, through calcium-mediated signaling and VEGF production, play a pivotal role in glioma-associated angiogenesis and tumor progression. In addition, research by Blank et al. revealed that GAMs, in collaboration with granulocytes, may facilitate angiogenesis through VEGF-independent mechanisms by releasing alternative angiogenic mediators, thereby contributing to resistance against conventional VEGF-targeted therapies (70). These findings underscore the necessity of in-depth mechanistic dissection of the interactions between GAMs and vascular remodeling processes within the tumor microenvironment.

### 3.3 Immunosuppressive conditioning of the glioblastoma tumor microenvironment

**Figure 1:**
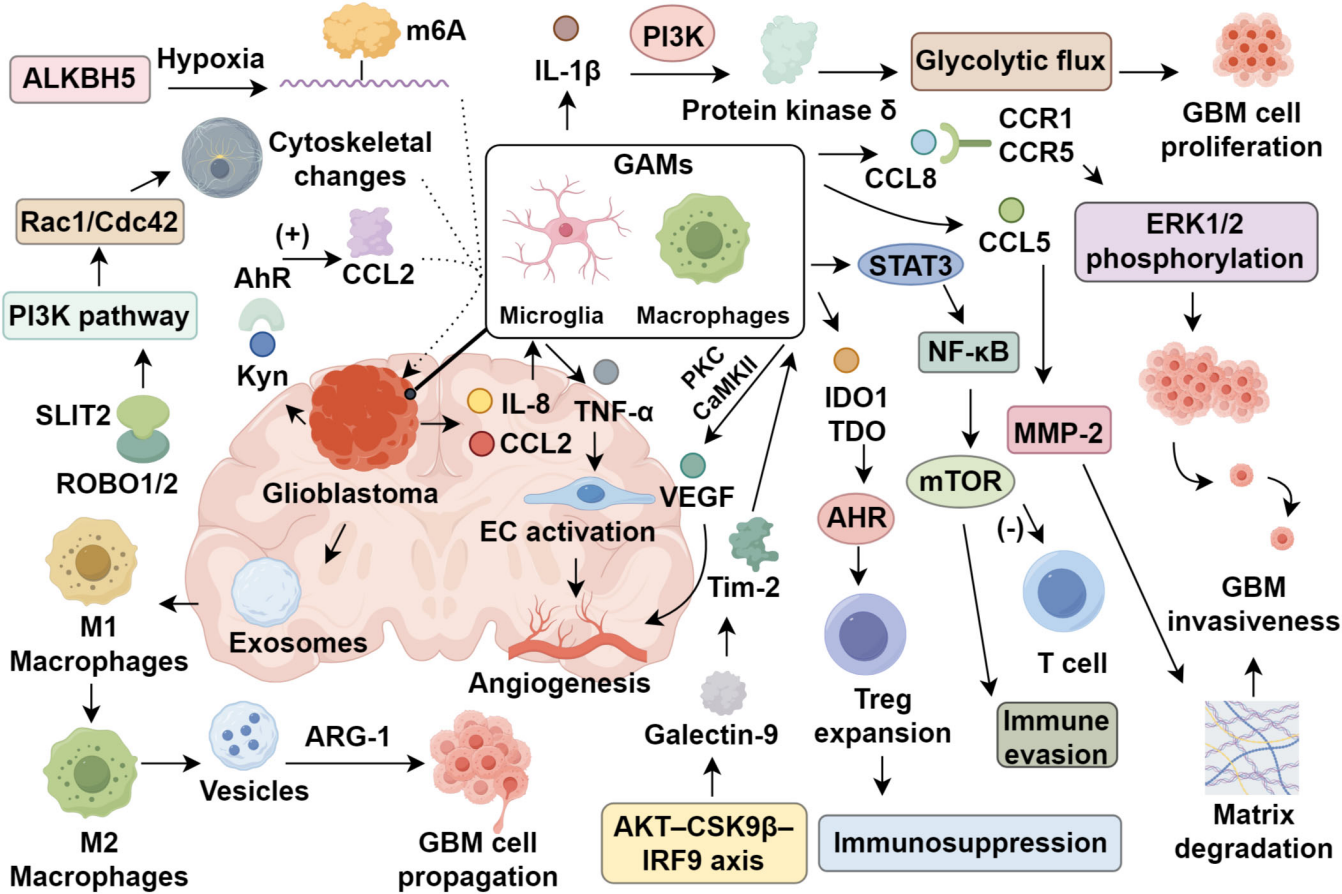
Glioma-associated microglia and macrophages in glioblastoma progression.

The establishment of an immunosuppressive tumor microenvironment is a critical mechanism by which glioblastoma circumvents immune detection and enables immune escape (43, 71). In GAMs, activation of the mammalian target of rapamycin (mTOR) signaling is driven through transcriptional programs orchestrated by STAT3 and NF-κB, culminating in a suppressive microglial phenotype that restricts the expansion, infiltration, and cytotoxicity of effector T lymphocytes, thereby promoting immune evasion (72). GAMs are a key cellular component of this immunosuppressive niche. Their heightened expression of indoleamine 2,3-dioxygenase-1 (IDO1) and tryptophan 2,3-dioxygenase (TDO) initiates the catabolism of tryptophan into L-kynurenine (L-Kyn), which activates the aryl hydrocarbon receptor (AHR). This promotes the expansion of regulatory T cells and the generation of tolerogenic myeloid populations, further reinforcing local immunosuppression (73–75). This metabolic reprogramming, orchestrated through the kynurenine pathway, facilitates tumor progression by modulating immune cell infiltration and cytokine secretion, creating a microenvironment that supports immune evasion and tumor growth (76–78). Additionally, glioblastoma cells accumulate quinolinic acid, a byproduct of the kynurenine pathway, which activates NMDA receptors and triggers the Foxo1/PPARγ axis in GAMs, driving their polarization towards tumor-promoting states (79, 80). In a study by Magri et al. (81), suppression of heme oxygenase-1 (HO-1) in microglia enhanced IL-10 production, concurrently inhibiting the STAT3/PD-L1 cascade and reducing IDO1 and ARG-2 transcription, collectively alleviating the immunosuppressive characteristics of the TME. Moreover, the phosphatase PP2A, in conjunction with its regulatory subunit STRN4, modulates Hippo signaling by dephosphorylating MST1/2 kinases, thus stabilizing the transcriptional co-activators YAP/TAZ. This regulatory circuit suppresses interferon-stimulatory gene (STING) signaling, further facilitating immune escape in glioblastoma (82). These multifaceted interactions underscore the importance of delineating the functional interplay between GAMs and the immunoregulatory landscape of the TME to refine and enhance the effectiveness of immunotherapeutic approaches in GBM (**Figure 1**).

## 4 Immunotherapy targeting GAMs

### 4.1 Targeting GAM recruitment and depletion in GBM

Interrupting the infiltration of GAMs has emerged as a viable strategy in the treatment of glioblastoma. Within the central nervous system, CCL2 serves as a critical chemokine mediating GAM (9, 83). In murine models of glioma, administration of a CCR2-specific antagonist alone extended median survival, while concurrent blockade with anti–PD-1 therapy yielded further survival gains, substantiating its potential for early-phase clinical evaluation (84). Osteopontin (OPN), a component of the extracellular matrix, plays a substantial role in attracting GAMs in a concentration-dependent manner within the tumor microenvironment of GBM (16, 85). Secreted by tumor cells, OPN interacts with GAMs surface receptors—particularly via CD44—to promote chemotaxis and sustain M2-like gene expression and cellular polarization. Inhibition or reduction of OPN production profoundly impairs the recruitment efficiency of the GAMs (85, 86). Further mechanistic insights from Chen et al. (87) revealed that in GBM models deficient in PTEN, the transcriptional coactivator YAP1 upregulates lysyl oxidase (LOX), whose secreted form activates the β1 integrin–PYK2 signaling axis to drive macrophage accumulation. Pharmacological suppression of LOX consequently attenuates macrophage infiltration and limits tumor advancement. Another regulator of the GAM landscape is the sodium–hydrogen exchanger (NHE), specifically the SLC9A1 isoform. Overexpression of this transporter correlates with heightened GAM density in the TME (88). In preclinical glioma models, inhibition of NHE1 using HOE642 dampens both macrophage infiltration and angiogenic processes, culminating in reduced tumor expansion (89). Despite GAMs being genomically stable and highly responsive to local environmental cues, efforts to eradicate these cells have faced challenges. Liposome-encapsulated clodronate, once internalized by phagocytes, induces programmed cell death (90). However, its intracerebral delivery depletes not only resident microglia but also inadvertently harms other neural and vascular components, indicating insufficient specificity in targeting GAMs (91). Therefore, such indiscriminate depletion strategies demand careful reconsideration due to their potential off-target effects.

### 4.2 Therapeutic repolarization of GAMs

A promising approach to curtail glioma advancement involves disrupting the M2 polarization state of GAMs and promoting a shift toward an M1-like phenotype (92, 93). Various agents—such as CSF-1R antagonists, chlorogenic acid, inhibitors targeting mTOR, lipopolysaccharide, curcumin-loaded phytosomes (CCP), and duloxetine—have demonstrated efficacy in redirecting GAMs toward an inflammatory, tumor-suppressive state, thereby mitigating their tumor-supportive properties (94–97). Central to this immunomodulatory process is the colony-stimulating factor 1 receptor (CSF-1R), whose blockade has been shown to reorient CD163^+^ macrophages away from an immunosuppressive state. Specifically, the CSF-1R inhibitor BIZ954 enhances glioblastoma responsiveness to radiotherapy and synergizes with anti–PD-1 immune checkpoint blockade, effectively augmenting antitumor immunity (98, 99). Nevertheless, the impact of CSF-1R inhibition is not uniform across glioblastoma subtypes. For instance, PLX3397 substantially inhibits PDGFB-driven gliomagenesis, yet paradoxically accelerates RAS-driven variants and exerts minimal influence on other proneural or mesenchymal tumor models. The mechanisms underlying these subtype-specific differences in response remain largely elusive (99). Additional repolarization strategies involving small interfering RNAs (siRNAs), microRNAs (miRNAs), or immunomodulatory cytokines face considerable translational barriers, including cytotoxicity, limited target specificity, and adverse systemic reactions, all of which constrain their clinical utility and warrant further optimization (100, 101). It is also critical to consider the unintended consequences of GAMs repolarization, as altering macrophage phenotypes may inadvertently suppress endogenous antitumor responses or induce systemic inflammatory side effects. A nanoparticle platform designed to deliver mRNA encoding interferon regulatory factor 5 (IRF5) *in vivo* (102). Upon cellular uptake, the mRNA engages IKKβ, its activating kinase, successfully redirecting GAMs toward a tumor-inhibitory phenotype while avoiding systemic toxicity (102, 103).

### 4.3 GAMs and oncolytic virotherapy

The therapeutic action of oncolytic herpes simplex virus (OHSV) involves not only direct lysis of tumor cells but also the activation of a long-lasting anti-tumor immune response (104, 105). Nevertheless, the host’s innate antiviral mechanisms frequently suppress viral replication within the tumor, thereby limiting oncolytic efficacy (106, 107). Among the key mediators of this suppression TNFα secreted by GAMs has been identified as a principal effector of apoptosis in virally infected cells, consequently diminishing intratumoral viral spread. Inhibition of TNFα markedly augments viral replication *in vivo* and correlates with enhanced therapeutic outcomes, as demonstrated in clinical investigations (108, 109). Additional mechanistic studies have revealed that phosphorylation of STAT1 and STAT3 impairs OHSV-1 propagation in GAMs (110). Notably, pharmacological blockade of STAT1/3 signaling using the oxindole/imidazole compound C16 facilitates increased viral replication in these immune cells, bolstering OHSV-1’s oncolytic potential against glioblastoma multiforme and promoting tumor regression (111).

## 5 Conclusion

Glioblastoma (GBM) presents a formidable clinical challenge, underscored by its rapid progression, immune evasion, and dismal prognosis. A defining feature of the GBM microenvironment is the extensive infiltration of GAMs, which orchestrate a spectrum of tumor-promoting functions—including metabolic rewiring, angiogenesis, and immunosuppression—while remaining largely unresponsive to current immunotherapeutic regimens. The dual origin of GAMs, their spatial–temporal heterogeneity, and their plasticity under dynamic cues confer both complexity and opportunity for targeted intervention.

Emerging strategies aimed at attenuating GAMs recruitment, depleting pro-tumor macrophages, or reprogramming their phenotypic state have shown preclinical promise. Moreover, modulation of GAMs signaling networks has demonstrated synergy with immune checkpoint inhibitors and oncolytic viruses, offering a rationale for combinatorial therapeutic regimens. Nonetheless, challenges remain in translating these findings into clinical benefit, including off-target effects, phenotypic rebound, and tumor subtype–specific resistance. Future efforts must prioritize precise GAMs subpopulation mapping, longitudinal tracking of their functional states, and development of delivery platforms that minimize systemic toxicity. Ultimately, targeting the immunological plasticity of GAMs may unlock new therapeutic potential in GBM and reshape the landscape of myeloid-based immunotherapy.
